# Prevalence of Autism Spectrum Disorder (ASD) among the children aged 18-36 months in a rural community of Bangladesh: A cross sectional study

**DOI:** 10.12688/f1000research.13563.1

**Published:** 2018-04-04

**Authors:** Shaheen Akhter, A.H.M Enayet Hussain, Jannatara Shefa, Gopen Kumar Kundu, Fazlur Rahman, Animesh Biswas

**Affiliations:** 1 Institute of Paediatric Neurodisorder and Autism (IPNA), Bangabandhu Sheikh Mujib Medical University, Dhaka, 1000, Bangladesh; 2 Non Communicable Disease Control (NCDC), Directorate General of Health Services, Dhaka, 1206, Bangladesh; 3 Centre for Injury Prevention and Research, Dhaka, 1206, Bangladesh; 4 Bangladesh University of Health Sciences, Dhaka, 1206, Bangladesh

**Keywords:** Autism spectrum disorder (ASD), children, prevalence, rural community, Bangladesh

## Abstract

**Background: **Autism spectrum disorder (ASD) refers to a group of complex neurodevelopment disorders characterized by repetitive and characteristic patterns of behavior and difficulties with social communication and interaction. In Bangladesh, autism in children is a significant burden of disease. Early identification of ASD could improve quality of life. The study has explored at the prevalence of ASD among rural community children aged between 18-36 months.

**Methods**: A cross sectional study was conducted among the 5286 children aged between 18-36 months in a rural community. Household level data was collected using screening tool MCHAT. Primarily screening positive 66 children were invited for final diagnosis in a health camp. Diagnosis was made by different staging started from primary screening, followed by validation using MCHAT and flash card. Final diagnosis was made by the paediatric neurologists, child clinical psychologists and development therapist using diagnostic tools (DSM-IV & ADOS).

**Results:** 04 children were diagnosed with autism spectrum disorder (ASD). Prevalence of the ASD in rural community was found 0.75/1000 children. Among the four ASD cases three were boys and one was girl and age range was between 20- 30 months. Whereas, the highest prevalence rate found was for the cerebral palsy which was 5.6/1000 children and Developmental delay (2.6/1000) was the next to that.

**Conclusions: **Age specific autism (18-36 months) in children is found higher in rural community of Bangladesh. In order to get more comprehensive information on autism in other age groups of children in rural community, further study is required. Early detection in rural community could help the policy makers to decentralization of health services among the ASD children in rural community.

## Introduction

In recent years, epidemiological studies have shown a rapid increase in the prevalence of autism spectrum disorders (ASD) ^1, 2^. Throughout the world, it is reported to be 1 in 150 children (See Centre for Research and Information site). According to estimates of the Centre for Disease Control and Prevention (CDC)’s Autism and Developmental Disease Monitoring (ADDM) Network, approximately 1 in 68 children aged 8 years are identified with ASD ^3^. California Department of Developmental Services (CDDS) and IDEA data sets are qualitatively consistent in suggesting a strong increase in autism prevalence over recent decades ^4^. Prevalence studies from European countries, with an age range of birth to adulthood, varied from 1.9/10000 to 72/10000 ^5^. A systematic review article reported differences in prevalence of autism in South Asia. It ranged from 0.09% in India to 1.07% in Sri Lanka ^6^

The disease manifests at an early age in children, and is likely to last for life ^7^. ASD severely affects the social functioning of an individual and may have a negative impact on the entire family of the affected individual ^1^. The accuracy of the numbers regarding prevalence of ASD depends on diagnostic criteria, age and geographical location, service availability and awareness of ASD ^8^. Advanced maternal and perinatal age is also a risk factor for ASD, with significantly increased risk with each 10-year increase in maternal age ^9^.

Average prevalence of ASD in Asia was 1.9/10000 before 1980, while it is 14.8/10000 from 1980 to present ^10^. The overall reported prevalence of ASD in recent studies was higher than previously reported in Asia.

In Bangladesh, it has been predicted that autism is an underestimated, yet significant health problem. In community studies done by Mullick and Rabbani in 2005 and 2009, autism was 0.2 and 0.84/1000 children respectively ^11, 12^. From a systematic review, the prevalence of ASD was found to be ranging from 0.15–0.8% in Bangladesh ^6^. Cambridge medical university’s patient registration records showed an increased rate of autistic children seeking treatment, from 12 children in 2001 to 105 children in 2009 ^13^. A national level study in Bangladesh in 2013 using a community level approach found prevalence of autism to be 0.15% amongst a population of 7200 in seven upazilas (Debhhata, Wazirpur, Pirgong, Godagari, Pekua, Madhupur and Kulaura and a city corporation ward of Dhaka city) ^14^. In another study by the ministry of Social Welfare, Bangladesh 2016, the proportion of autism was found to be 19% of total neurological disabilities recorded ^15, 16^.

It is essential to identify the cases of ASD as early as possible because educational planning and initiation of interventions results in better outcomes for these children ^17– 19^. There are different methodologies applied in different studies to identify prevalence in Bangladesh. However, age specific prevalence of autism is not yet determined in Bangladesh. This study has explored the age specific (18–36 months) ASD prevalence among children from a rural community of Bangladesh.

## Methods

A cross sectional study was conducted during the period of April 2016 to June 2016.

### Study area

Raiganj upazila (sub-district) of Sirajganj district is located in the northern part of Bangladesh. The upazila consists of nine unions (small unit of upazila) with a population of more than 300,000. The study implementing organization Center for Injury Prevention and Research, Bangladesh (CIPRB) has its own ongoing surveillance system functioning in six unions of this upazila. Thus, the study has chosen rural community of all six unions for the study.

### Study population

A total number of 255,265 populations reside in 55,492 households of the six unions of Raiganj upazila in Sirajganj district. All households of the selected unions were included for this study.

### Sampling

**Figure 1.**
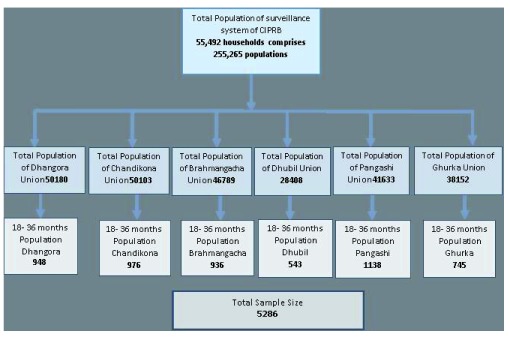
Framework of sampling technique.

The study selected all children aged from 18 months to 36 months. Their information was recruited from the household database of the six unions’ surveillance system. A total number of 5600 children were identified from the surveillance data base. All households of those children were selected for collection of data at the household level ( Figure 1).

**Expected outcome:** The study looked for autism spectrum disorder as outcome and exposure was children of age 18–36 months either boys and girls in rural community.

### Data collection

Fifteen field level survey data collectors were recruited. Three supervisors were assigned to supervise and monitor the survey data collectors throughout the process of data collection and to confirm positive screened cases at the household level. All field level staff received one-day comprehensive training on data collection, which was conducted by a team of paediatric neurologists, public health specialists and child development therapists. The data collectors and supervisors were trained on using pictorial flash cards, which they were instructed to use during the time of interview to collect desired information (examples of flash cards are available in Supplementary File 1).

The data collectors performed face to face interviews with the mother at the household level using a structured questionnaire ( Supplementary File 2). M-CHAT (Modified Checklist for Autism in Toddlers) was used as the primary screening tool for ASD. The survey data collectors used pictorial flash cards for each of the 23 questions of M-CHAT to let the mother understand if their children have similar type of manifestations as shown in the pictorial flash cards.

A total number of 98 primarily screened positive cases were listed by the survey data collectors using M-CHAT. Out of 5600 children, the data collectors were able to collect data from 5286 children aged between 18 months to 36 months. Remaining families had either migrated from the unions or were not found during the data collection period. No respondent refused to provide information. The study has included participants those who were identified as M-CHAT positive during screening at the households to invite in the health camp.

### Data quality assurance and confirmation of MCHAT positive cases

Three supervisors visited the 98 households after initial identification of the positive screened cases by the survey data collectors. The same M-CHAT and flash cards were used by the supervisors to confirm the primary diagnosis. Out of 98 primary positive screened cases, 68 cases were identified as M-CHAT positive cases. 30 cases were primarily screened out as MCHAT positive by the data collectors, did not match with the M-CHAT criterion during the 2 ^nd^ visit by the supervisors. Before the exclusion of the 30 cases, investigators checked the MCHAT collected by the data collectors and the supervisors through a discussion meeting. Finally, 68 positive screened families were invited with their children to attend the medical camp for final diagnosis. Health camp was organized at CIPRB’s Raiganj research field office located in Dhangora union on 31 ^st^ May 2016. Additionally, the investigators were involved in the process of quality assurance throughout the whole procedure of participation in trainings, data collection. They also randomly selected 5% of positive cases to check the consistency of data collection.

**Bias and confounders:** The study identified bias in identification of M-chat positive by the field level data collectors using pictorial flash cards. Initially, 98 children were identified as M CHAT positive. To prevent potential bias, all 98 children with positive M CHAT were visited by skilled supervisors. 68 cases finally identified M-CHAT positive, remains which were found M Chat negative, the supervisors sit with the research investigators to come up on final discussion to exclude from the study. The study did not address for any confounders.

### Instruments used for diagnosis

I. **M-CHAT:** It is a validated tool for assessing the risk of ASD in screening toddlers aged between 16 to 36 months. The M-CHAT can be administered and scored as part of a well-child check-up, and also can be used by specialists or other professionals to assess risk for ASD. Users need to be aware that even with the follow-up questions, a significant number of the children who fail the M-CHAT will not be diagnosed with an ASD. However, these children are at risk for other developmental disorders or delays, and therefore, evaluation are warranted for any child who fails the screening. Children who fail more than 3 items total or 2 critical items has been identified as initial screening positive for further diagnostic evaluation by professional experts to evaluate ASD in very young children ^20, 21^.
II. **DSM- IV TR:** Diagnostic and Statistical Manual of Mental Disorders (DSM-IV TR), published by the American Psychological Association, is the standard for the classification of mental disorders. As ASD became known throughout the United States, and common symptoms and behaviors were agreed upon by many researchers, it gained increasingly specific diagnostic criteria in the DSM. Here, autism is traced throughout the four main domains of the DSM ^22^.
III. **ADOS:** The Autism Diagnostic Observation Schedule (ADOS) is an instrument for diagnosing and assessing autism. It became commercially available in 2001 through the Western Psychological Services (WPS). The protocol consists of a series of structured and semi-structured tasks that involve social interaction between the examiner and the subject. The examiner observes and identifies segments of the subject's behavior and assigns these to predetermined observational categories. Categorized observations are subsequently combined to produce quantitative scores for analysis ^23^.
IV. **Flash card:** Twenty-three pictorial flash cards were developed based on 23 questions for M-CHAT to use in this study. All flash cards were drawn in a pictorial format. Flash cards were used during the survey at the household and shown each of the sign/symptoms in a pictorial form for better understanding for the mothers.

### Diagnosis of ASD and other neurodevelopmental disorders (NDDs)

**Figure 2.**
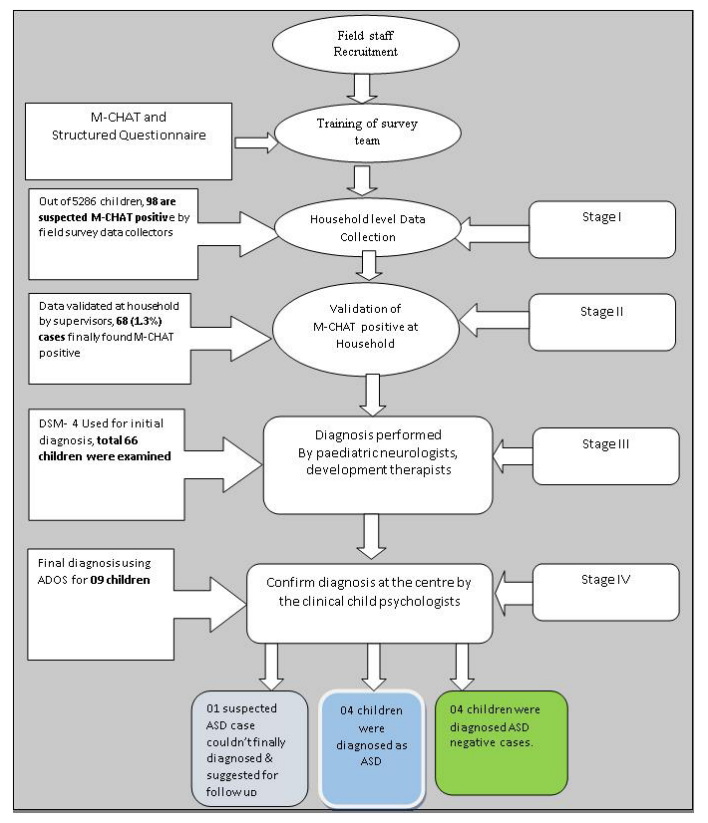
Methodological framework.

A medical camp was organized at the upazila level to confirm the diagnosis and management of affected children. Out of M-CHAT positive 68 children, 66 children with their parents came to health camp for diagnosis. A team of two paediatric neurologists, three medical doctors experienced in working with children with autism, two child clinical psychologists, one development therapist from Institute of Paediatric Neurodisorder and Autism (IPNA), Bangabandhu Sheikh Mujib Medical University (BSMMU) along with a public health specialist (epidemiologist) conducted the health camp. The diagnosis and treatment were performed on three groups of children. The first group were all diagnosed with the DSM-4 TR, suspected ASD positive cases were then sent to the child clinical psychologist group for confirmation of ASD using the ADOS test. Finally, the third group provided management for all children including those who were identified as ASD or other NDDs ( Figure 2).

### Management and referral

Three medical doctors, including one senior doctor, were involved in providing treatment for the children according to the diagnosis made by the paediatric neurologists. Management includes prescribing medicine, counseling, provision of physical therapy and emergency referral to a nearby referral centre of the district or IPNA of BSMMU.

### Data analysis

SPSS version 20 for windows was used for descriptive analysis. The prevalence and the confidence intervals were calculated using the software EPI Info Version 6.04d.

### Ethical clearance

Ethical permission for this study was obtained from the Institutional Review Committee, Centre for Injury Prevention and Research, Bangladesh [Memo: CIPRB/ERC/2016/009]. Participants of the study were children aged between 18–36 months, to obtain details information about the children, written consent was obtained from the each of the parents to participate and provide information.

## Results

The majority of the mothers were aged between 18 and 30 years (74.2%). Most of them were had a low level of education or had no formal education (56.1%). The majority of the children were diagnosed between the age of 31 and 36 months (41%). Boys had found higher prevalence of diseases then girls (65% vs 55%) [ Table 1].

**Table 1.** Characteristics of rural mothers and children in the study.

Characteristics of mother and child	Percentage (%)
*Age of the mothers*	
18 – 30 years	83.3 (n=55)
31 – 35 years	9.0 (n=6)
31 – 45 years	7.5 (n=5)
*Mother’s education*	
Illiterate	9.1 (n=6)
Up to primary education	47.0 (n=31)
Up to secondary education	39.4 (n=26)
Up to higher secondary education	4.5 (n=3)
*Child age*	
18 – 24 months	33.0 (n=22)
25 – 30 months	26.0 (n=17)
31 – 36 months	41.0 (n=27)
*Sex of child*	
Boys	65.0 (n=43)
Girls	35.0 (n=23)
*M-CHAT*	
Positive	62.0 (n=41)
Negative	38.0 (n=15)
*ADOS*	
Yes	13.6 (n=9)
No	86.4 (n=57)

**Figure 3.**
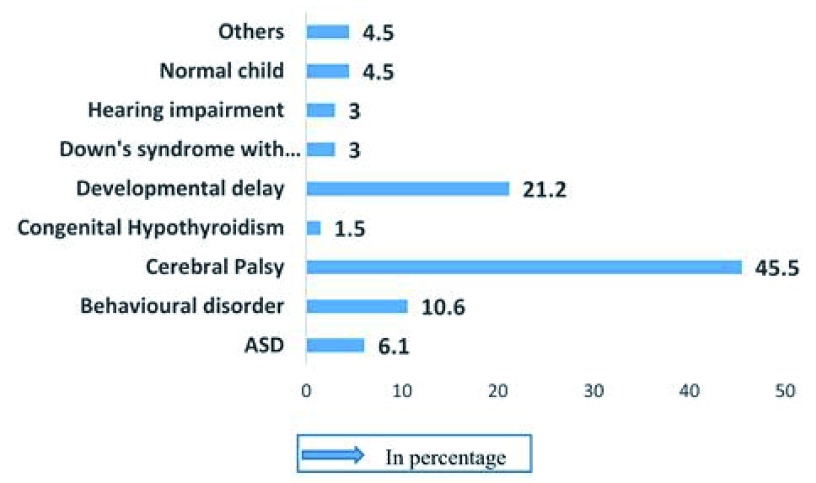
Diseases diagnosed among the suspected cases (n=66).

Among the 66 children of rural community of Raiganj uapzila, three children were found to with no disease (4.5%). Several children were identified to have cerebral palsy (45.5%), whereas autism was diagnosed in only 6.1% of cases (n=4). Developmental delay was found as the second highest disease amongst the children (21.2%) ( Figure 3).

### Prevalence of ASD and other neurological disorders

**Figure 4.**
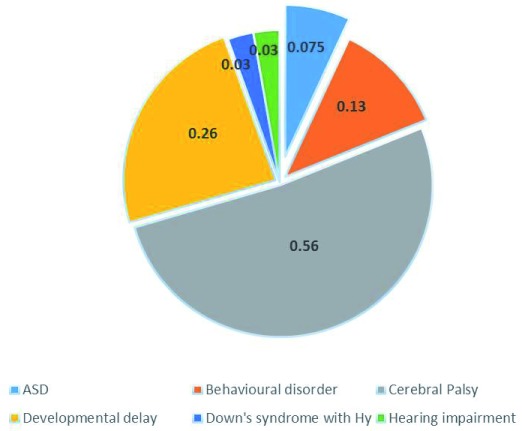
Prevalence of ASD (in percentage) and other neurological disorders.

From the total sample (n=5286), only 4 cases were found to have ASD with a prevalence of 0.75/1000 children. Among the four cases three were boys and one was a girl, and their age ranged from 20–30 months. The highest prevalence rate found was for cerebral palsy at 5.6/1000 children. Developmental delay (2.6/1000) was the second highest prevalence ( Figure 4).

### Heath service delivery

**Figure 5.**
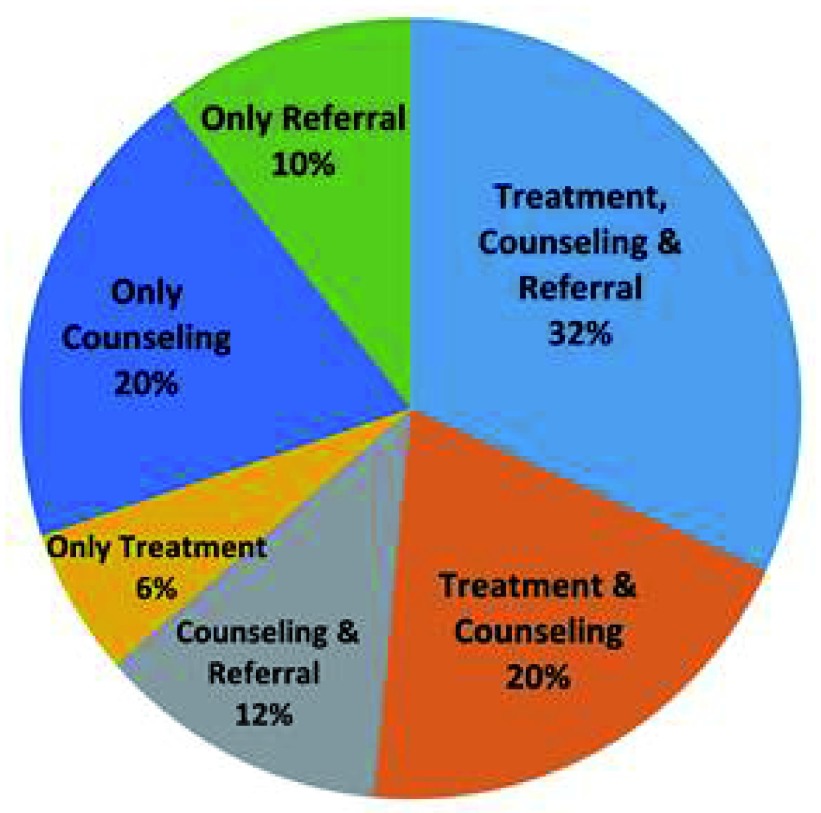
Treatment, counseling and referral of the suspected children.

Out of 66 children, three children were diagnosed with no disease and no treatment or advice was suggested for them. For the remaining 63 children, 10% of the children were directly referred to the specialized centre for further management. A combination of medication, counseling and referral were advised for around 32% of children ( Figure 5).

## Discussion

The study found that the prevalence of autism is 0.75/1000 in rural children aged between 18–36 months, with prevalence of cerebral palsy and developmental delays being much higher. Age specific early diagnosis of autism in the rural community was not explored earlier. A national study conducted in 2013 showed that the overall prevalence of ASD was 1.55/1000 amongst the study group of 7280 children aged between 0–9 years. The study also revealed that prevalence of ASD in the rural community studied was 0.68/1000 ^14^. Although it was estimated that about 300,000 children were affected with autism in Bangladesh with one case in every 94 boys, and one in every 150 girls, was estimated to suffer from ASD (see Autistic Children’s Welfare Foundation site). These findings are consistent with the present study. In the study by ministry of Social welfare of Bangladesh prevalence came out as 3% of total population. But in the study all the age groups were included ^15^.

This study examined early detection of autism in children considering that the major symptoms of autism can be identified at this age group (2–3 years), and proper management as well as improved quality of life can be achieved from early detection ^24^. ASD is usually described as a childhood neuro-developmental disorder with onset usually before 3 years of age ^25^.

This study found that 56.1% of the mothers of ASD suspected children had low level or no formal education background. Another study also found the highest prevalence of ASD amongst children whose mothers only attended primary schools ^26^. Contrary to this one study found children of parents with a higher educational background had a higher prevalence of childhood autism ^27^.

This study also revealed that 74.2% mothers of suspected cases were aged between 18 to 30 years, with mothers with an early age of pregnancy had the higher rate of autistic children. This is in contrast with the study where advancing maternal age had found to be associated with higher risk of autism ^28^. We also found autism to be more prevalent in boys than girls (65% vs 55%). A study in the US also revealed similar findings, where one in 42 boys and one in 189 girls were found with ASD ^29^.

A number of good examples were found on the estimation of prevalence of autism from the perspective of developed countries. Some countries used DSM-III for diagnostic purposes whereas others used ADOS ^30, 31^. Bangladesh has trialed for the first-time a community based system in the detection of autism among rural and urban communities through three stages of data collection, processing, and confirmation of cases ^15^. This study used a community based population level survey at the household using pictorial flip cards to screen the cases for the first time. The study also used a four-stage data collection and confirmed diagnosis using a community based health camp. The study also followed the pathway of diagnosis to management, which creates a boarder spectrum of benefits for the early detection of autism and other neuro developmental diseases, as well as referral to the higher centers like ’child development center’ run by the Government of Bangladesh in different Medical Colleges and IPNA, BSMMU. Moreover, during management, each of the parents were counseled by the doctors who could help to improve overall quality of care for those children with autism and other neuro developmental disorders.

It is alarming that other neuro developmental diseases like cerebral palsy and developmental delays are many folds higher than autism. It is important to do further research on other NDDs so that special interventions can be designed based on the findings.

The study has been done at the sub district level within a confined population which may not be representative of the country. For better understanding of the real magnitude of the problem, a larger study is required, in a bigger population considering early age detection of ASD and other NDDs. Move over, it is also important to do further community based studies using the new approach adopted for primary diagnosis.

The country is well ahead for the reduction of under-five and infant mortality rate ^32^. The country also has a very structured community level health infrastructure and primary health care delivery system, where community clinics have delivered health care services to people’s doors. It is time to respond on early detection of the ASD and other NDDs using the primary health care model. Although the definite causes of ASD are genetic, environmental modification and improvement of the quality for health care will improve the overall situation. Like this, early detection of ASD and NDDs will help the parents and their family to take immediate care for their better healthy quality of life.

## Data availability

The study involved multiple stakeholders including government, professional organizations and research institute. Data is stored at the CIPRB and in Institute of Paediatric Neurodisorder and Autism (IPNA), Bangabandhu Sheikh Mujib Medical University (BSMMU), Dhaka. Due to sensitivity of the data (contains identifying information), permission is required from the ethical committee for sharing data with a third party. Data requests should be sent to Institute of Paediatric Neurodisorder and Autism (IPNA), Bangabandhu Sheikh Mujib Medical University (BSMMU), Dhaka who will contact the ethical review committee to gain approval to share the data. The conditions for gaining data access are a formal request with a clear objective and formal permission from the ethical committee. Please contact the Prof. Shaheen Akhter, Institute of Paediatric Neurodisorder and Autism (IPNA), Bangabandhu Sheikh Mujib Medical University (BSMMU), Dhaka through email : shaheenk33@gmail.com in order to request the data.

## Supplementary material

**Supplementary File 1 – Zip file contain examples of the flash cards used in this study**

**Supplementary File 2 – Study questionnaire (with English translation)**
