# Supplementary material for: Prevalence of Autism Spectrum Disorder (ASD) among the children aged 18-36 months in a rural community of Bangladesh: A cross sectional study
Source: F1000Res. 2018 Apr 4;7:424. [Version 1] doi: 10.12688/f1000research.13563.1 (PMC6039957; doi:10.12688/f1000research.13563.1)
Supplement: Peer review history

# Peer review: Referee response for version 1

**DOI:** 10.5256/f1000research.14732.r35553

**Published:** 2018-06-28

**Authors:** Soumyadeep Bhaumik

**Affiliations:** 1 The George Institute for Global Health, New Delhi, Delhi, India

This manuscript intends to estimate the prevalance of autism spectrum disorder in rural children of 18-36 months.It uses a cross-sectional study design for this purpose. Comments for consideration are :

 1.The use of the word age-specific in the objective is not neessary in my opinion.

 2.  The manuscript would benefit if it follows the STROBE reporting guidelines for cross-sectional study. Such reporting will enable understanding of the methodology better for the purpose of critique and reproducibility. This is mandatory as per F1000 guidelines.

 3. Please clarify if the criteria for selection of unions - was it all the rural unions in Raiganj Upazila or was it the functioning ongoing surviellence system. Are the three unions which were not included as rural classified as urban by the government ? It might be good to name the six unions included.

 4. The manuscript mentions all households of selected union was included - how was the list of universe of households obtained ?

 5. Not sure what the expected outcome sub-section within sampling is for - consider deletion.

 6. Please provide reference to MCHAT anf flash card  methods being used in this study in other similar study. I am not sure why DSM-IV TR or ADOS is explained in the manuscript . Consider discussing why the issues are different in different studies.

 7.Explain how the study size was calculated - was it powered for gender disaggregation. Was it powered for measuring other neurological conditions described in the paper. The result thus provides many other things and there is need of clarity to understand this better.

 9. Discussion needs a section on potential limiations of the study and ways that has been taken to address the bias. How do the results of the study compare to that in other SEARO countries.

 10. The Abstract conclusion mentions  " Age specific autism (18-36 months) in children is found higher in rural community of Bangladesh. " It is not clear higher in comparison to what ? The idea about needing more studies in future is generic. Please consider providing more specific information.

 11. Some points mentioned in the discussion like the "56.1% of the mothers of ASD suspected children had low level or no formal education background. "  are probably of no intepretive value considered this is not a RR or OR and the fact that only 4 cases were found. Same for other things in the discussion.

I have read this submission. I believe that I have an appropriate level of expertise to confirm that it is of an acceptable scientific standard, however I have significant reservations, as outlined above.

# Peer review: Referee response for version 1

**DOI:** 10.5256/f1000research.14732.r35443

**Published:** 2018-06-27

**Authors:** Helen McConachie

**Affiliations:** 1 Institute of Health and Society, Newcastle University, Newcastle upon Tyne, UK

This cross-sectional study makes an estimate of the prevalence of autism spectrum disorder in very young children (18-36 months) in Bangladesh. The study utilised the opportunity of an ongoing rural surveillance system to carry out door to door screening using the M-CHAT tool enhanced by the use of flash-cards. Screen-positive status was checked by supervisors, and around two -thirds confirmed. These children were invited for diagnostic assessment, including where indicated assessment using a gold-standard diagnostic tool, the Autism Diagnostic Observation Schedule. A prevalence estimate of 0.75/1000 was concluded.

 The paper cites the most relevant literature, including a 2013 Survey of Autism and Neurodevelopmental Disorders in Bangladesh conducted for the Directorate General of Health Services, Ministry of Health and Family Welfare, Government of Bangladesh. That survey found a very similar prevalence estimate of ASD in rural children of 0.68/1000. It is therefore incorrect for the authors to state in the Abstract: "Age specific autism (18-36 months) in children in found higher in rural community of Bangladesh". The estimate is low in comparison globally.

 The methodology adopted for the study appears strong in the main. However, a prevalence estimate with confidence intervals would require that a random sample of children screened negative were also invited for further assessment.  Some of the interpretation of results is also inappropriate as statements are made, for example, about the numbers of children identified with cerebral palsy, without clearly stating that this estimate is within only the screened positive and assessed children.

 The authors' conclusions are encouraging, that systems for early detection and management of neurodevelopmental disorders can now be used across Bangladesh, because of good primary health care and the establishment of Child Development Centres in the Medical College Hospitals.

I have read this submission. I believe that I have an appropriate level of expertise to confirm that it is of an acceptable scientific standard, however I have significant reservations, as outlined above.

# Peer review: Referee response for version 1

**DOI:** 10.5256/f1000research.14732.r32768

**Published:** 2018-04-18

**Authors:** Olalekan A. Uthman

**Affiliations:** 1 Warwick Medical School, Health Sciences Research Institute, University of Warwick, Coventry, UK

The authors of the manuscript conducted a cross-sectional study to estimate the prevalence of Autism Spectrum Disorder among children in Bangladesh. The manuscript has potential to contribute to the body of knowledge in the field. The authors should be congratulated on bringing to light this important, but often neglected spectrum of medical conditions in resource-limited settings. The manuscript reads well and conclusion stated were not beyond the findings of this cross-sectional study.

 I have no major concerns in the conduct and reporting of this study:

1. Abstract: ‘04’ should be changed to word at the start of that sentence.
2. Table 1: column two, consider reporting the number and percentage in the bracket and change the column heading to ‘Number (%)’
3. Discussion: Consider adding study strengths and limitations
4. Discussion: In addition, consider adding sub-heading to the discussion section, such as ‘Main findings, ‘Implications for policy and research’, ‘Study strengths and limitations’
5. Conclusion: consider also adding conclusion at the end of the discussion section

I have read this submission. I believe that I have an appropriate level of expertise to confirm that it is of an acceptable scientific standard.
